# Coenzyme Q10 protects against atorvastatin-induced hepatotoxicity via attenuation of oxidative stress and functional modulation of CYP3A1

**DOI:** 10.1186/s40360-026-01125-z

**Published:** 2026-04-09

**Authors:** Mohammed A. Abdel-Rasol, Mohamed S. Anwar, Hoda A. Taha, Wael M. El-Sayed, Kholoud AbdelRaouf

**Affiliations:** Department of Zoology, Faculty of Science, Ain Shams University, Abbassia, Cairo, 11566 Egypt

**Keywords:** Apoptosis, Drug-induced liver injury, Drug-metabolizing enzymes, Nuclear receptors, Oxidative stress

## Abstract

**Background:**

Atorvastatin (ATO) is a widely prescribed lipid-lowering drug, but its use can be limited by hepatotoxicity, potentially linked to metabolism-related mechanisms. The cellular pathways connecting ATO metabolism to oxidative imbalance and apoptotic alterations remain incompletely defined. This study examined the roles of oxidative stress, mitochondrial-associated apoptosis, and the pregnane X receptor (PXR)–CYP3A4 axis in ATO-induced hepatic changes, and evaluated whether coenzyme Q10 (CoQ10) could modulate these effects depending on administration timing.

**Methods:**

Forty male albino rats were randomly assigned to four groups (*n* = 10). Group I received vehicle (5% DMSO + olive oil), Group II received atorvastatin (ATO, 80 mg/kg), Group III received ATO (80 mg/kg) plus CoQ10 (10 mg/kg), and Group IV received ATO for 21 days followed by CoQ10 for 21 days. All treatments were administered orally once daily. Hepatic outcomes were assessed using biochemical markers, histopathology, and Bcl-2 immunohistochemistry. Microsomal CYP3A1 (rat ortholog of human CYP3A4) catalytic activity was determined using a testosterone 6β-hydroxylation assay. Molecular docking evaluated interactions of ATO, its lactone metabolite, and CoQ10 with CYP3A4 and PXR.

**Results:**

ATO induced oxidative imbalance, evidenced by increased lipid peroxidation and nitric oxide alongside depleted antioxidant defenses, accompanied by liver structural alterations and reduced Bcl-2 expression. ATO significantly reduced hepatic microsomal CYP3A1 activity to ~ 29% of vehicle control (~ 71% decrease). CoQ10 co-administration markedly attenuated these effects, restoring redox homeostasis and increasing Bcl-2 expression, while co-treatment restored CYP3A1 activity (~ 90% of vehicle control). Post-treatment with CoQ10 partially restored CYP3A1 activity (~ 60% of vehicle control). Docking analysis indicated favorable binding of ATO and its lactone to CYP3A4 and PXR, supporting metabolism-linked bioactivation, while CoQ10 exhibited lower affinity, suggesting indirect modulation.

**Conclusion:**

ATO-induced hepatic injury is associated with oxidative stress and mitochondrial-related apoptotic processes linked to metabolism-dependent mechanisms. Functional impairment of CYP3A1 activity accompanied ATO-induced oxidative damage, whereas CoQ10 preserved enzymatic activity, consistent with hepatocellular protection. Early CoQ10 intervention provided superior protection, highlighting the importance of timing in mitigating metabolism-related hepatic damage. These findings provide mechanistic insight into statin-induced hepatotoxicity and support further investigation of CoQ10 as a complementary strategy to enhance statin safety.

## Introduction

Drug-induced liver injury (DILI) represents a persistent clinical and regulatory challenge, as it can progress to hepatic failure and remains a common cause of drug withdrawal [1]. Statins are among the most widely prescribed medications for reducing cardiovascular risk through inhibition of 3-hydroxy-3-methylglutaryl-coenzyme A (HMG-CoA) reductase, the rate-limiting enzyme in cholesterol biosynthesis [2]. Atorvastatin (ATO), in particular, is extensively used in clinical practice; however, it is also one of the statins most frequently associated with hepatotoxic effects [3]. This coexistence of well-established therapeutic benefit and dose-limiting adverse effects has driven continued interest in adjunct strategies aimed at improving statin safety, particularly those targeting mitochondrial stability and oxidative balance, such as coenzyme Q10 (CoQ10).

ATO is subject to extensive hepatic metabolism, primarily via cytochrome P450 3A4 (CYP3A4), resulting in the formation of hydroxylated metabolites as well as ATO lactone [4]. These metabolic products are not simply inactive bystanders but can actively contribute to hepatic injury. This process is further amplified by activation of the pregnane X receptor (PXR), which induces CYP3A4 expression and thereby enhances ATO bioactivation. In the present study, CYP3A1, the rat ortholog of human CYP3A4, was measured as a functional indicator of this metabolic pathway. Consequently, the same metabolic pathway responsible for drug clearance may also increase the formation of reactive intermediates, placing hepatocytes under sustained metabolic and oxidative stress [5–7].

At the downstream level, oxidative stress plays a central role in the development of ATO-associated hepatotoxicity. Reactive oxygen and nitrogen species (ROS and RNS) generated during ATO metabolism damage cellular lipids, proteins, and DNA, disrupt mitochondrial function, and trigger apoptotic signaling pathways [8, 9]. At the same time, endogenous antioxidant defenses—including glutathione (GSH), catalase (CAT), and superoxide dismutase (SOD)—are rapidly depleted, further increasing cellular susceptibility to injury [10]. Under physiological conditions, mitochondrial integrity is maintained by anti-apoptotic proteins such as Bcl-2, which prevent cytochrome c release and downstream caspase activation [11]. Oxidative stress compromises this protective mechanism, shifting the balance toward Bax-mediated mitochondrial membrane permeabilization and apoptosis [12]. Taken together, these events suggest that ATO-induced hepatotoxicity arises from an interconnected cascade involving drug metabolism, oxidative imbalance, and mitochondrial-driven cell death.

CoQ10 has attracted attention as a potential modulator of this cascade. Beyond its established function in the mitochondrial electron transport chain, CoQ10 contributes to stabilization of mitochondrial membranes, limits opening of the mitochondrial permeability transition pore (mPTP), and supports cellular bioenergetics [13]. Its antioxidant properties enable scavenging of ROS, preservation of redox homeostasis, and attenuation of oxidative injury. By maintaining mitochondrial integrity, CoQ10 may also suppress apoptotic signaling and thereby reduce ATO-induced hepatic damage [14, 15].

Despite advances in understanding the mechanisms underlying ATO-associated hepatotoxicity, several important questions remain unresolved. In particular, the extent to which PXR-driven CYP3A4 activation contributes to mitochondrial dysfunction and apoptosis has not been fully defined. In addition, the influence of intervention timing has received limited attention, raising the question of whether prophylactic CoQ10 administration during ATO exposure provides greater hepatoprotection than therapeutic supplementation initiated after injury onset.

To address these gaps, the present study investigated the effects of CoQ10 on ATO-induced hepatic alterations, with specific emphasis on the PXR–CYP3A4 axis, oxidative stress, and apoptotic signaling. By integrating in vivo experimental approaches with molecular docking analyses, and including functional CYP3A1 measurements in rats, this work aims to clarify mechanistic pathways linking ATO metabolism to hepatic vulnerability and to evaluate CoQ10 as a prophylactic or therapeutic adjunct to improve statin safety.

## Materials and methods

### Chemicals

Atorvastatin (Atrostat^®^ 40 mg; El Delta Pharmaceutical Industries, Egypt) and CoQ10 (Coenzyme Q10^®^ 10 mg; Arab Company for Pharmaceuticals and Medicinal Plants, Cairo, Egypt) were used in this study. Unless otherwise specified, all reagents were purchased from Sigma-Aldrich (St. Louis, MO, USA). Commercial kits for ALT (Cat. No. 264 001), total (Cat. No. 222 001) and direct bilirubin (Cat. No. 222 001), T. chol. (Cat. No. 230 001), TAG (Cat. No. 314 001), HDL-chol. (Cat. No. 267 001), and creatinine (Cat. No. 234 001) were obtained from Spectrum Diagnostics (Cairo, Egypt). All reagents and kits were of analytical grade and handled according to the manufacturers’ protocols.

### Experimental animals

Adult male albino rats (150 ± 20 g) were obtained from the National Research Center (Dokki, Egypt). Animals were acclimatized for one week before experimentation and maintained under controlled housing conditions (temperature 25 ± 1 °C and relative humidity 60 ± 5%) with a 12 h light/dark cycle. Rats had *ad libitum* access to water and standard rodent chow. All experimental procedures were approved by the Institutional Ethics Committee, Faculty of Science, Ain Shams University (ASU-SCI/ZOOL/2025/11/01) and conducted in accordance with the Guide for the Care and Use of Laboratory Animals. Animals were observed daily for clinical or behavioral changes, and any signs of illness were recorded.

### Experimental design

**Fig. 1:**
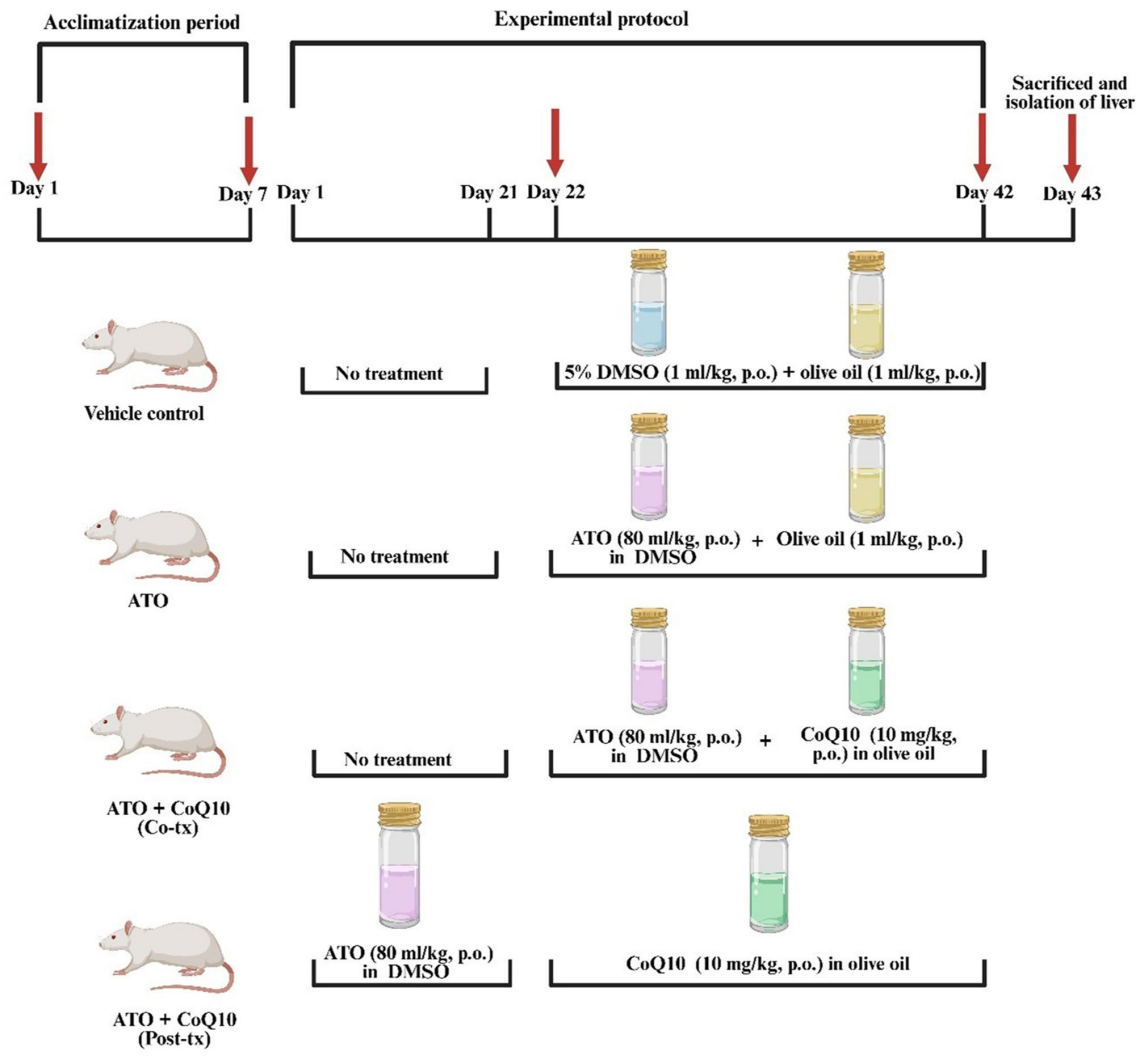
Schematic diagram of the experimental design to evaluate prophylactic and therapeutic CoQ10 regimens in mitigating ATO-induced hepatotoxicity. Abbreviations: ATO, atorvastatin; CoQ10, coenzyme Q10; Co-tx, cotreatment; Post-tx, post-treatment; p.o., oral administration

The study was conducted over 42 days, during which 40 male albino rats were randomly assigned to four groups of 10 animals each. All groups underwent a standard 7-day acclimatization period before the start of the experiment. Groups I–III were then given an additional 21 days of acclimatization before the initiation of treatments on day 22, whereas Group IV began treatment immediately from day 1, following the standard 7-day acclimatization period. Group I served as the vehicle control and received 5% DMSO (1 mL/kg) combined with olive oil (1 mL/kg) by oral gavage once daily for 21 days, starting on day 22. Group II was administered ATO at a dose of 80 mg/kg dissolved in 5% DMSO, followed by olive oil (1 mL/kg), once daily for 21 days, beginning on day 22 [16]. Group III received a combination of ATO (80 mg/kg in 5% DMSO) and CoQ10 (10 mg/kg in olive oil, 1 mL/kg), given orally once daily for 21 days starting on day 22 [17]. Group IV was treated with ATO (80 mg/kg in 5% DMSO) orally once daily for the first 21 days, after which treatment was immediately followed by CoQ10 (10 mg/kg in olive oil, 1 mL/kg) once daily for an additional 21 days, extending to day 42 (Fig. 1).

### Blood sampling and tissue extraction

At the end of the experiment, rats were anesthetized with ketamine (100 mg/kg) and xylazine (5 mg/kg) intraperitoneally and humanely euthanized by cervical decapitation to collect blood. Blood was collected into sterile tubes, allowed to clot at room temperature, and centrifuged at 1789 × g for 15 min at 4 °C. Serum was aliquoted and stored at -80 °C for biochemical analyses.

Livers were perfused via the portal vein with ice-cold isotonic saline, excised, blotted, and weighed. A portion of the left lateral lobe was fixed in 10% formalin for histopathology and immunohistochemistry. The remaining tissue was snap-frozen at -80 °C for biochemical assays. For homogenization, 1 g of liver tissue was homogenized in 10 mL of ice-cold phosphate buffer saline (PBS, pH 7.4). Homogenates were centrifuged at 11,180 × g for 20 min at 4 °C, and the supernatant was stored at -80 °C for assays of oxidative and antioxidant markers.

The remaining liver tissue was homogenized in cold 0.1 M potassium phosphate buffer (pH 7.4) supplemented with 250 mM sucrose and 1 mM EDTA. The homogenate underwent an initial centrifugation at 10,000 × g for 20 min at 4 °C to remove nuclei, mitochondria, and cell debris. The supernatant was then subjected to ultracentrifugation at 105,000 × g for 60 min at 4 °C to isolate the microsomal fraction. The resulting microsomal pellet was resuspended in storage buffer (0.1 M potassium phosphate, pH 7.4, 20% glycerol, 1 mM EDTA/Dithiothreitol) and kept at -80 °C until further use in enzyme activity assays. Protein concentration was determined using the Folin-Ciocalteu phenol method.

### Biochemical assays in liver

Markers of oxidative stress and antioxidant defense in liver homogenates were evaluated by quantifying MDA, NO, GSH, CAT, and SOD. MDA levels were determined by reaction with thiobarbituric acid (TBA), producing a chromogenic MDA-TBA adduct whose absorbance was measured at 532 nm; concentrations were calculated against a standard curve prepared with tetraethyl pyridine (TEP) [18]. NO was assessed using the Griess reaction, which forms a pink chromophore measurable spectrophotometrically, with concentrations interpolated from a sodium nitrite standard curve [19]. GSH content was measured by its reaction with 5,5’-Dithiobis (2-nitrobenzoic acid) (DTNB), yielding a yellow-colored product recorded at 412 nm [20]. CAT activity was quantified by monitoring the decomposition of Hydrogen Peroxide (H_2_O_2_) at 240 nm, using the molar extinction coefficient of 43.6 M/cm [21]. SOD activity was measured by its ability to inhibit nitroblue tetrazolium (NBT) reduction in the xanthine–xanthine oxidase system, with absorbance read at 560 nm [22]. Results were normalized to protein content and expressed as units per mg protein.

### Histopathological examination

Formalin-fixed liver tissue (*n* = 5 biological samples per group) was processed through graded ethanol, cleared in terpineol, embedded in paraffin wax, and sectioned at 5 μm thickness. Sections were deparaffinized, rehydrated, and stained with H&E. Slides were mounted and examined under a Leica DM750 microscope, and images were captured using a Leica ICC50 HD camera. Liver injury was scored semi-quantitatively on a 0–3 scale (0 = absent, 1 = mild, 2 = moderate, 3 = severe) [23].

### Immunohistochemical analysis

Sections (*n* = 5 biological samples per group) mounted on charged slides were deparaffinized, rehydrated, and treated with 10% H_2_O_2_ to quench endogenous peroxidase activity. Antigen retrieval was carried out in sodium citrate buffer (pH 6.0) at 90 °C for 20 min. Non-specific binding was blocked with 5% bovine serum albumin (BSA) in tris-buffered saline (TBS). Slides were incubated with a polyclonal rabbit anti-Bcl-2 antibody (1:100) (Cat No. PA5-27094; Thermo Fisher Scientific, WA, USA) overnight. After washing, sections were incubated with a peroxidase-conjugated goat anti-rabbit secondary antibody (BioCare Medical, Walnut Creek, CA, USA). Signal was visualized with 3,3’-diaminobenzidine DAB/H_2_O_2_ and counterstained with Harris hematoxylin. Images were acquired on a Leica DM750 microscope with a Leica ICC50 HD camera. For quantification, Bcl-2 positive staining was analyzed using ImageJ software. The relative area covered by positive staining was measured for each section and expressed as a percentage of the total section area. This quantification allowed for statistical comparisons between the different groups.

A subset of 5 animals per group was used for histopathology and immunohistochemistry due to tissue processing constraints, consistent with standard practice.

### CYP3A1 activity

Microsomal CYP3A1 activity was evaluated by preparing a reaction mixture containing 900 µL of 100 mM potassium phosphate buffer (pH 7.4), 20 µL of 1 mM NADPH, and 50 µL of the microsomal fraction, with the final volume adjusted to 1 mL using the same buffer. The reaction was initiated by the addition of NADPH and incubated at 37 °C for 10–20 min to allow enzymatic conversion of testosterone (10 µM) to 6β-hydroxytestosterone. The product was quantified fluorometrically at an excitation wavelength of 350 nm and emission of 450 nm [24]. Specificity of the reaction was confirmed using ketoconazole as a selective CYP3A1 inhibitor in control assays.

### Molecular modelling

Crystal structures of CYP3A4 (PDB ID: 1W0E) and PXR (PDB ID: 1M13) were retrieved from the Protein Data Bank. Structures were prepared with AutoDock Tools v1.5.6 by removing water molecules, heteroatoms, and extra chains. Missing residues were modeled, and polar hydrogens and Kollman charges were added before conversion to pdbqt format. Ligand structures (ATO, ATO lactone, CoQ10, and reference ligands) were downloaded from PubChem in SDF format, energy-minimized using the MM94 force field, assigned Gasteiger charges, and converted to pdbqt format.

Grid boxes were defined at the binding sites of the co-crystallized ligands: CYP3A4 (center coordinates X = − 19.076, Y = − 23.927, Z = − 12.607) and PXR (X = 4.684, Y = 69.194, Z = 2.2), with dimensions of 40 Å in all directions. Docking was performed in AutoDock. Binding affinities were recorded in kcal/mol. The top three binding modes were analyzed, and the most relevant interaction with key residues was selected for discussion [25].

### Statistical analysis

The Shapiro-Wilk test was used to assess data normality. Data are presented as mean ± standard deviation (SD). Significant differences between groups were evaluated using one-way analysis of variance (ANOVA) followed by Tukey’s multiple comparison test. Statistical significance was set at a p-value of less than 0.05. A sample size of 10 animals per group was used, which was based on ethical considerations and available resources. Power calculations indicated that this number is slightly below the ideal for detecting moderate effect sizes (Cohen’s f ≈ 0.35) with 80% power and α = 0.05, but it is sufficient to identify trends and provide preliminary insights.

## Results

### CoQ10 attenuates atorvastatin-induced oxidative stress in liver tissue

**Fig. 2:**
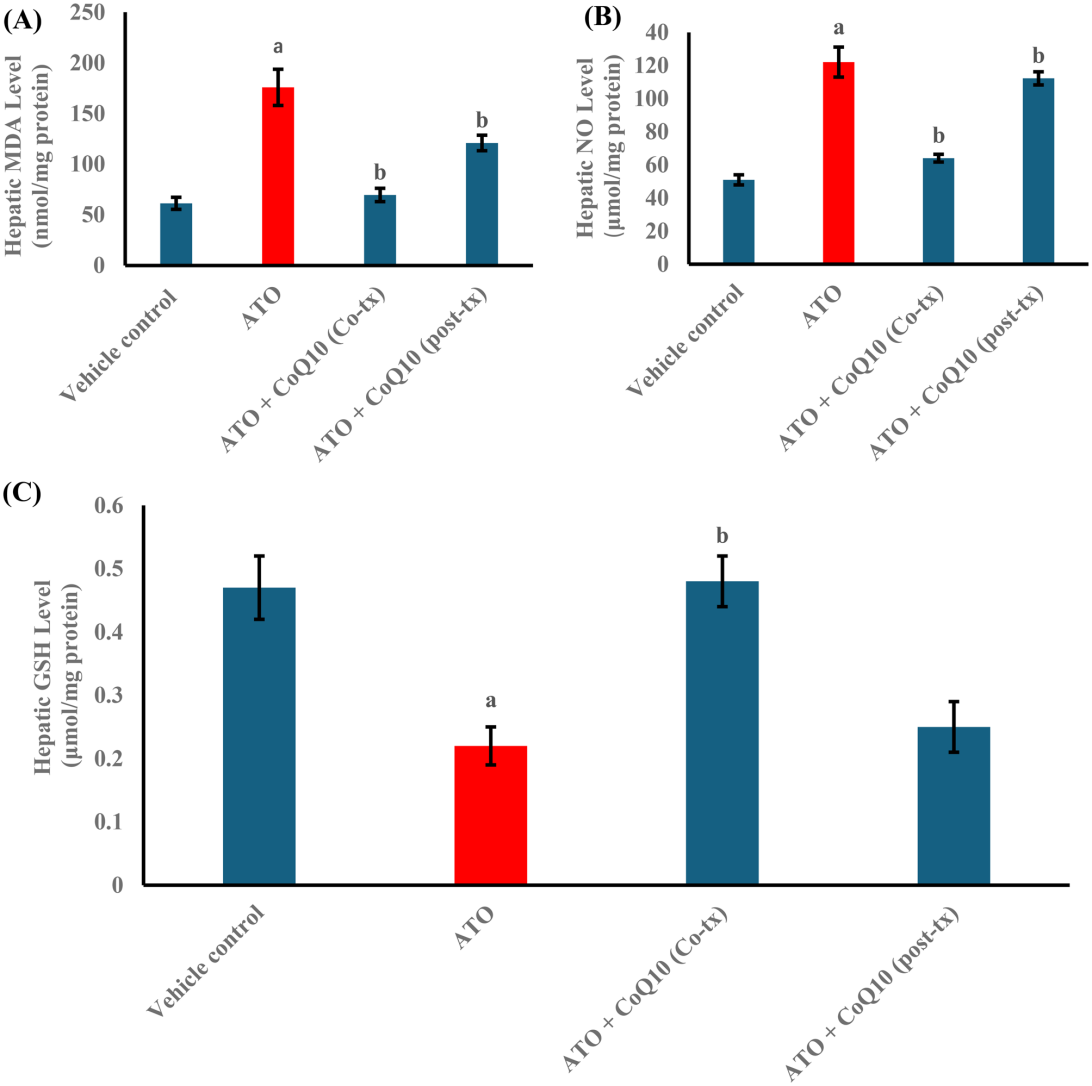
Effect of CoQ10 on hepatic levels of (**A**) MDA, (**B**) NO, and (**C**) GSH in atorvastatin-intoxicated male rats. Results are expressed as Mean ± SD (*n* = 10). Statistical significance was assessed using one-way ANOVA followed by Tukey’s post hoc test. ^**a**^ significant difference vs. Vehicle control (*p* < 0.05). ^**b**^ significant difference vs. ATO-treated group (*p* < 0.05). Abbreviations: MDA, malondialdehyde; NO, nitric oxide; GSH, reduced glutathione; ATO, atorvastatin; CoQ10, coenzyme Q10; Co-tx, cotreatment; Post-tx, post-treatment

Atorvastatin (ATO) markedly elevated oxidative stress markers. Hepatic MDA levels increased by 186% compared with the vehicle control, while NO rose by 140%. CoQ10 attenuated these effects, with co-treatment reducing MDA by 60% and NO by 48%, whereas post-treatment achieved more modest reductions of 46% and 26%, respectively (Fig. 2A, B).

**Fig. 3:**
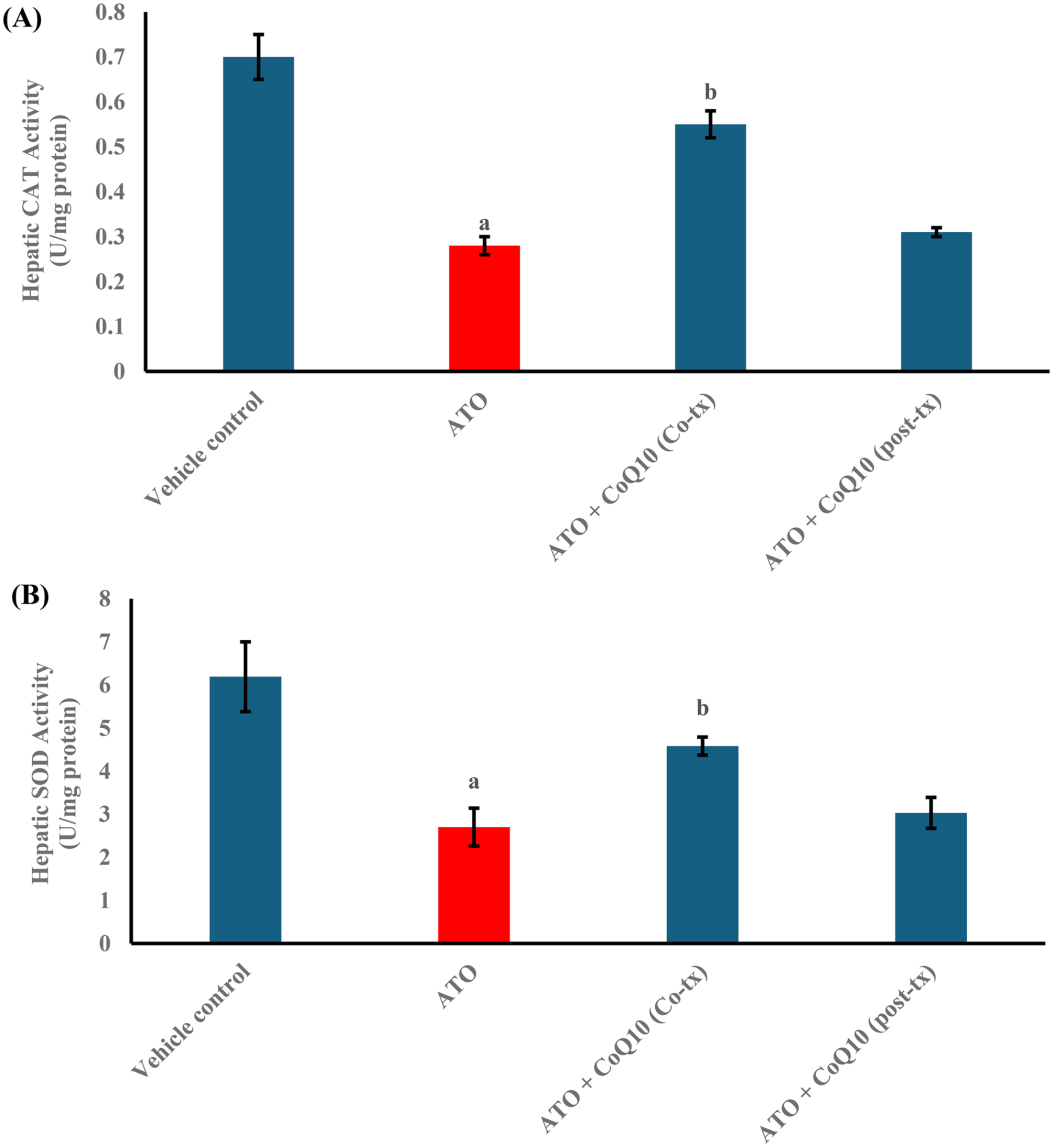
Effect of CoQ10 on Hepatic Activity of (**A**) catalase (CAT) and (**B**) superoxide dismutase (SOD) in Atorvastatin-Intoxicated Male Rats. Results are expressed as the Mean ± SD, *n* = 10. Statistical significance was assessed using one-way ANOVA followed by Tukey’s post hoc test. ^**a**^ significant difference vs. vehicle control (*p* < 0.05). ^**b**^ significant difference vs. ATO-treated group (*p* < 0.05). Abbreviations: ATO: Atorvastatin, CoQ10: Coenzyme Q10, Co-tx: Cotreatment, Post-tx: Post-treatment

In parallel, ATO suppressed antioxidant defenses. Hepatic GSH decreased by 53%, while CAT and SOD activities fell by 60% and 56%, respectively. CoQ10 co-treatment produced robust recovery, restoring GSH by 118%, CAT by 96%, and SOD by 70% compared with ATO alone. Post-treatment produced milder improvements, with increases of 14%, 11%, and 12% in these markers, respectively (Figs. 2C and 3A, B).

Taken together, these results demonstrate a consistent pattern: oxidative stress was markedly exacerbated by ATO, while co-treatment with CoQ10 provided stronger protection than post-treatment. These oxidative changes were mirrored in the subsequent functional biomarkers of hepatic and renal integrity.

### CoQ10 co-treatment restores liver and kidney function biomarkers in atorvastatin-treated rats

Functional assays confirmed that ATO significantly impaired liver and kidney function. ALT activity rose by 50%, direct bilirubin by 332%, total bilirubin by 556%, and serum creatinine by 202% relative to vehicle control. CoQ10 co-treatment substantially mitigated these elevations, reducing ALT, direct bilirubin, total bilirubin, and creatinine by 34%, 82%, 84%, and 47%, respectively. Post-treatment also improved function, though less effectively, with reductions of 25%, 61%, 54%, and 39% relative to ATO alone (Table 1).

**Table 1:** Effect of CoQ10 on liver and kidney function biomarkers, lipid profile, and hepatic microsomal CYP3A1 in ATO-intoxicated male rats

Treatment	Vehicle control	ATO	ATO + CoQ10 (Co-tx)	ATO + CoQ10 (Post-tx)
ALT (IU/L)	73.79 ± 0.72	110.42 ± 9.77^**a**^	72.92 ± 1.06^**b**^	83 ± 3.85^**b**^
D bil (mg/dL)	0.10 ± 0.03	0.42 ± 0.12^**a**^	0.09 ± 0.01^**b**^	0.16 ± 0.04^**b**^
T bil (mg/dL)	0.24 ± 0.05	1.58 ± 0.41^**a**^	0.25 ± 0.08^**b**^	0.72 ± 0.14^**b**^
Creatinine (mg/dL)	0.49 ± 0.03	1.48 ± 0.18^**a**^	0.79 ± 0.18^**b**^	0.90 ± 0.03^**b**^
T. chol. (mg/dL)	65.70 ± 12.75	45.10 ± 6.65^**a**^	69.30 ± 3.21^**b**^	79.70 ± 3.00^**b**^
TAG (mg/dL)	109.50 ± 11.01	29.00 ± 7.64^**a**^	35.88 ± 5.09	41.44 ± 13.47
HDL-chol. (mg/dL)	9.40 ± 0.44	23.82 ± 2.77^**a**^	23.94 ± 1.50	25.46 ± 2.57
LDL-chol. (mg/dL)	40.57 ± 11.90	14.47 ± 7.37^**a**^	38.24 ± 3.46^**b**^	42.28 ± 1.23^**b**^
CYP3A1 (nmol/min/mg protein)	0.89 ± 0.04	0.26 ± 0.02^**a**^	0.80 ± 0.03^**b**^	0.53 ± 0.05^**b**^

Results are expressed as the Mean ± SD, *n* = 10. ^**a**^ significant difference vs. vehicle control group. ^**b**^ significant difference vs. ATO group. Abbreviations: ALT, alanine aminotransferase; D bil, direct bilirubin; T bil, total bilirubin; T. chol., total cholesterol; TAG, triacylglycerol; HDL-chol., high-density lipoprotein cholesterol; LDL-chol., low-density lipoprotein cholesterol; ATO, atorvastatin; CoQ10, coenzyme Q10; Co-tx, cotreatment; Post-tx, post-treatment

Lipid metabolism was also disrupted by ATO. T. chol., TAG, and LDL-chol. were decreased by 31%, 74%, and 64%, while HDL-chol. increased by 153%. CoQ10 partially reversed these alterations, with post-treatment producing slightly stronger rebounds than co-treatment (Table 1). Interestingly, this pattern was consistent with the antioxidant findings, where co-treatment showed greater normalization of oxidative markers, while post-treatment offered partial but weaker protection.

### CoQ10 improves liver histopathological changes induced by atorvastatin

**Fig. 4:**
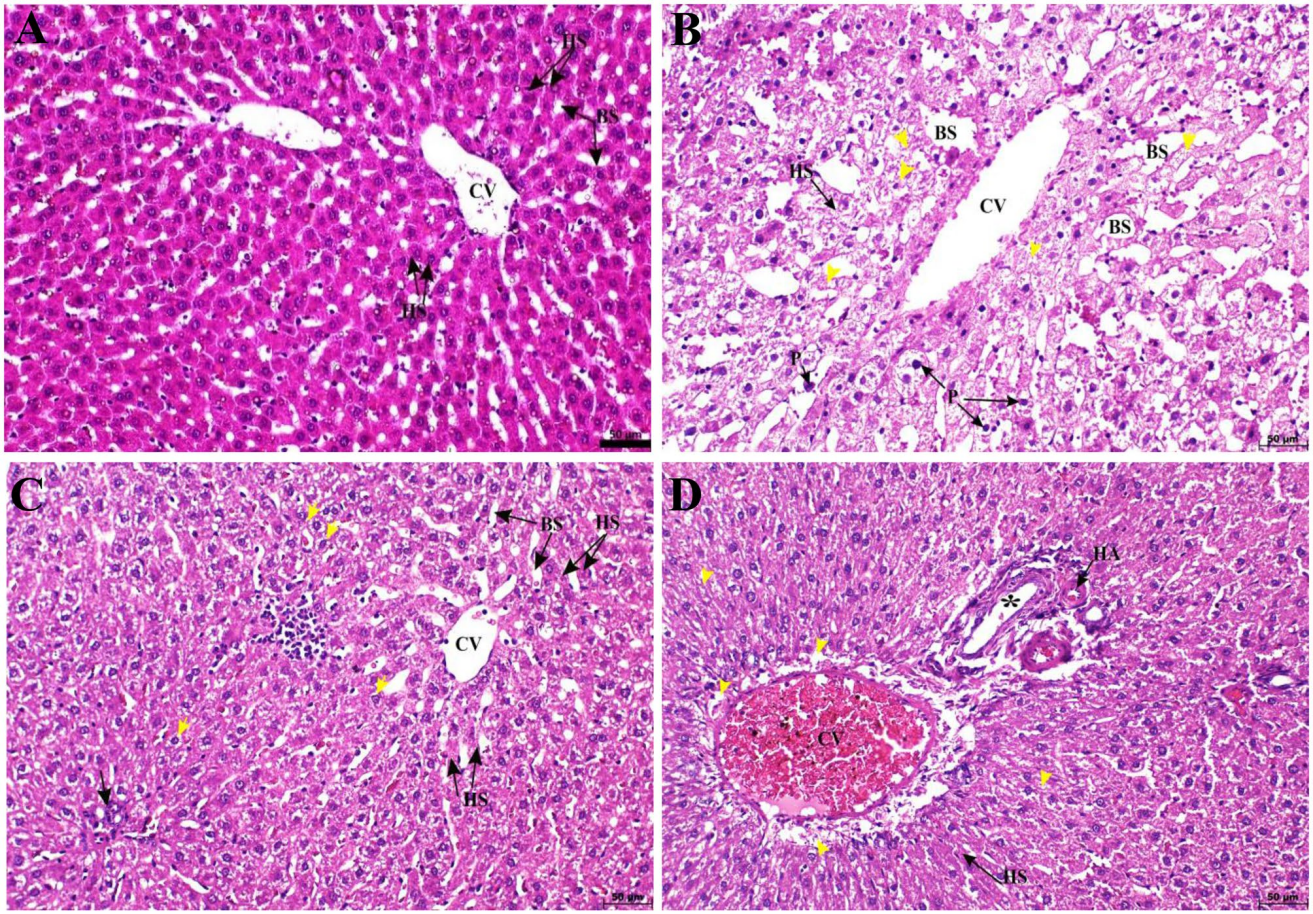
Photomicrographs of liver sections stained with H&E. (**A**) vehicle control group showing intact hepatic architecture with a central vein (CV) surrounded by well-arranged hepatic strands (HS) and normal blood sinusoids (BS). (**B**) ATO-intoxicated group showing dilated CV, markedly distended BS, disrupted HS, hepatocellular vacuolation (yellow arrows), and pyknotic nuclei (P). (**C**) ATO + CoQ10 (Co-tx) group showing reduced hepatocellular vacuolation (yellow arrows), partially preserved HS, and Kupffer cell infiltration (black arrows) around the CV. (**D**) ATO + CoQ10 (Post-tx) group showing congested CV, HS, and bile duct (*) with degenerative cuboidal epithelium; scattered hepatocellular vacuolation (yellow arrows) is also evident. Abbreviations: CV, central vein; HS, hepatic strands; BS, blood sinusoids; ATO, atorvastatin; CoQ10, coenzyme Q10; Co-tx, cotreatment; Post-tx, post-treatment. Three fields were analyzed from each of the 5 biological samples per group (*n* = 5)

Histological evaluation further reinforced the biochemical findings. Vehicle control liver tissues showed intact architecture, with radially arranged hepatic cords, narrow sinusoids, and hepatocytes of normal morphology (Table 2; Fig. 4A). In contrast, ATO-treated rats exhibited severe injury: central vein enlargement, sinusoidal dilation, vacuolated hepatocytes, and disrupted lobular structure with numerous pyknotic nuclei. Histopathological scoring reflected this severity, with lesions graded as 3 (severe) for central vein dilation, sinusoidal dilation, vacuolation, and strand disruption (Table 2; Fig. 4B).

**Table 2:** Semi-quantitative histopathological scoring of liver tissue alterations

Parameter	Vehicle control	ATO	ATO + CoQ10 (Co-tx)	ATO + CoQ10 (Post-tx)
Central vein dilation	1	3	1	2
Central vein congestion	0	1	1	3
Sinusoidal dilation	0	3	1–2	2
Hepatocellular vacuolation	0	3	1	2
Disruption of hepatic strands	0	3	1	2
Pyknotic hepatocyte	0	2–3	1	2
Bile duct changes	0	0	0	2–3
Kupffer cell infiltration	0	0	2	1

Scores were assigned as follows: 0 = absent, 1 = mild, 2 = moderate, 3 = severe. Three fields were examined from each of the five biological samples. Abbreviations: ATO, atorvastatin; CoQ10, coenzyme Q10; Co-tx, cotreatment; Post-tx, post-treatment

CoQ10 co-treatment reduced vacuolar degeneration and better preserved hepatic cords, with lower scores (1 = mild) for sinusoidal dilation and vacuolation, though Kupffer cell infiltration was observed (score 2 = moderate) (Table 2; Fig. 4C). Post-treatment also showed recovery but remained incomplete: the central vein was dilated, and bile duct injury was evident, scoring 2–3 (moderate–severe) (Table 2; Fig. 4E). These gradings (1 = mild, 2 = moderate, 3 = severe) highlight the relative protective advantage of co-treatment over post-treatment.

### CoQ10 restores Bcl-2 expression in liver tissue after atorvastatin-induced damage

**Fig. 5:**
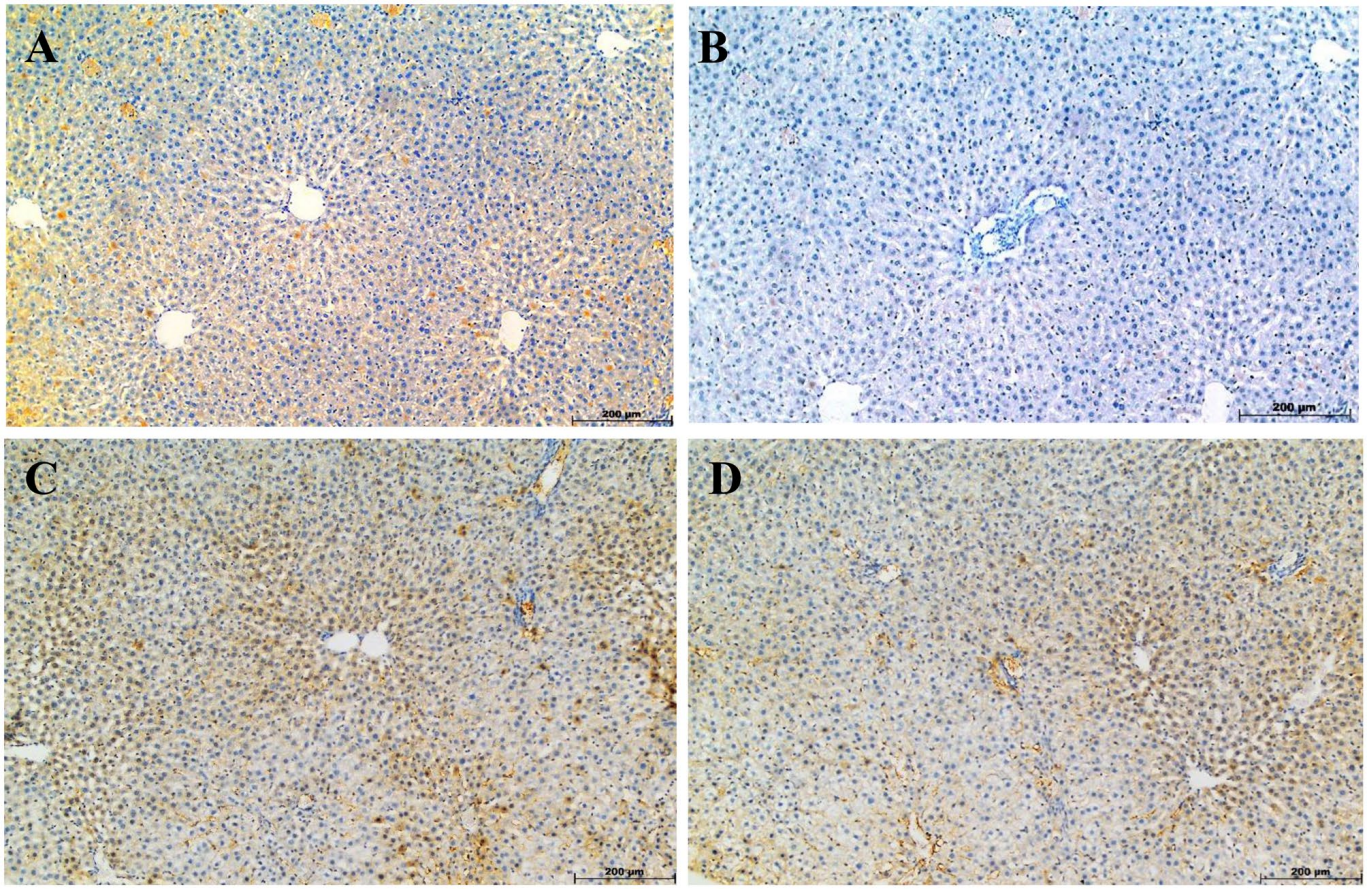
Photomicrographs of immunohistochemical staining for Bcl-2 expression in liver sections of rats. (**A**) Vehicle control group; (**B**) ATO-treated group; (**C**) ATO + CoQ10 (Co-tx) group; (**D**) ATO + CoQ10 (Post-tx) group. Intense Bcl-2 immunoreactivity is evident in the control, Co-tx, and Post-tx groups, whereas the ATO-treated group shows markedly reduced Bcl-2 staining. Three fields were analyzed from each of the five biological samples per group. Abbreviations: ATO, atorvastatin; CoQ10, coenzyme Q10; Co-tx, cotreatment; Post-tx, post-treatment

**Fig. 6:**
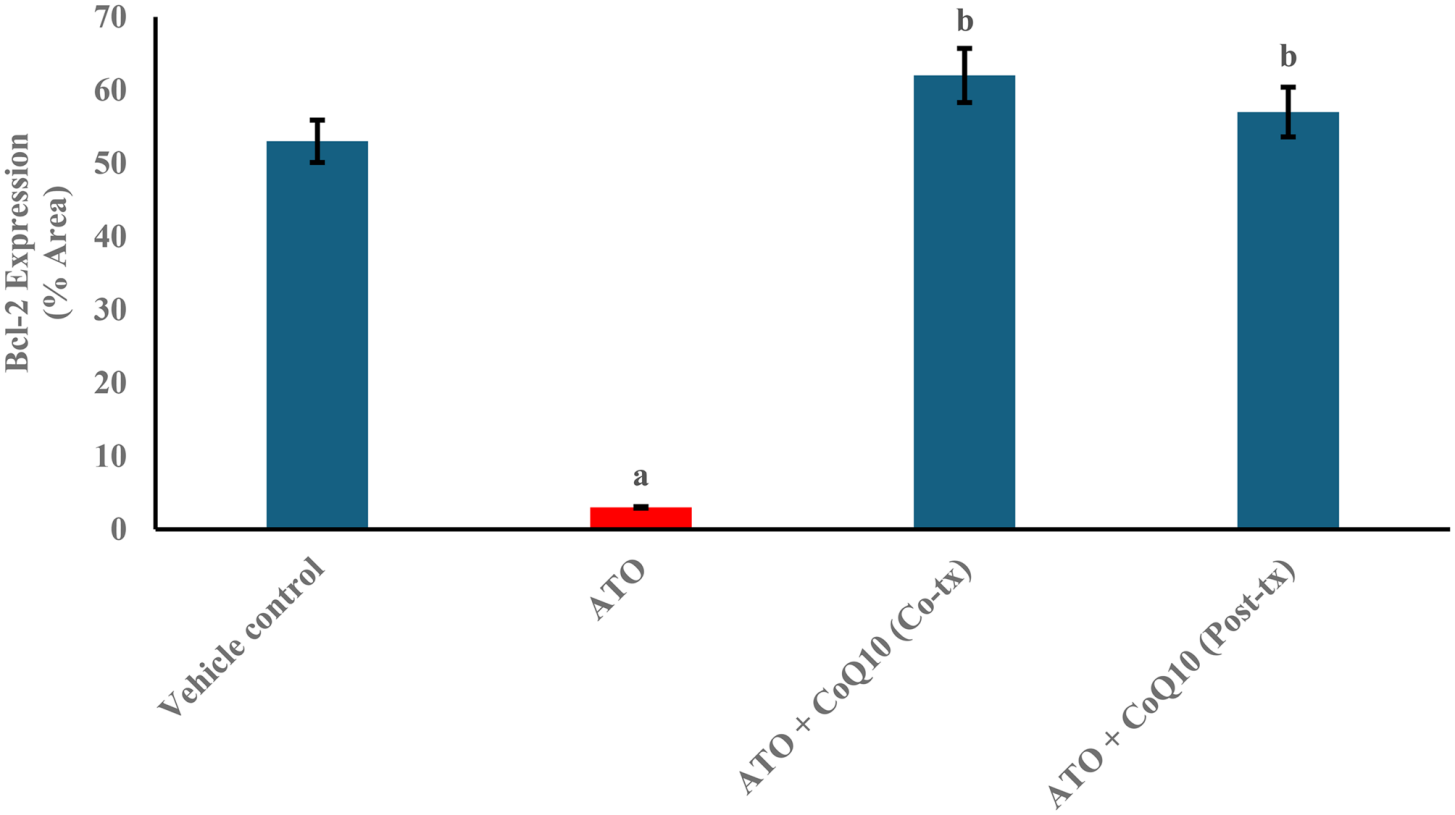
Quantification of Bcl-2 immunohistochemical expression in liver sections from different treatment groups. Results are expressed as Mean ± SD of the relative area covered by the positive reaction in reference to the whole section area (*n* = 5). Statistical significance was assessed using one-way ANOVA followed by Tukey’s post hoc test. ^**a**^ significant difference vs. vehicle control (*p* < 0.05). ^**b**^ significant difference vs. ATO-treated group (*p* < 0.05). Abbreviations: ATO, atorvastatin; CoQ10, coenzyme Q10; Co-tx, cotreatment; Post-tx, post-treatment

Bcl-2 immunoreactivity showed distinct variations among groups. Strong staining was observed in the vehicle control group (Figs. 5A and 6). ATO treatment reduced Bcl-2 expression by 94.34% compared with the vehicle control (Figs. 5B and 6). CoQ10 co-treatment increased Bcl-2 expression by 1966.7% relative to ATO alone (Figs. 5C and 6), while post-treatment produced an 1800% increase (Figs. 5D and 6). Both CoQ10 regimens markedly elevated Bcl-2 levels, with co-treatment showing the greater response. Notably, this pattern of co-treatment superiority mirrored the trends seen in oxidative stress markers, functional biomarkers, and histopathological scores.

### CYP3A1 activity and molecular docking reveal CoQ10’s potential interaction with CYP3A4 and PXR in atorvastatin metabolism

Hepatic microsomal CYP3A1 activity was significantly reduced (~ 71%) by ATO treatment (Table 1). Co-administration of CoQ10 largely preserved CYP3A1 activity (~ 90% of vehicle control), whereas post-treatment with CoQ10 partially restored activity (~ 60% of vehicle control), consistent with its comparatively weaker protective effect.

**Fig. 7:**
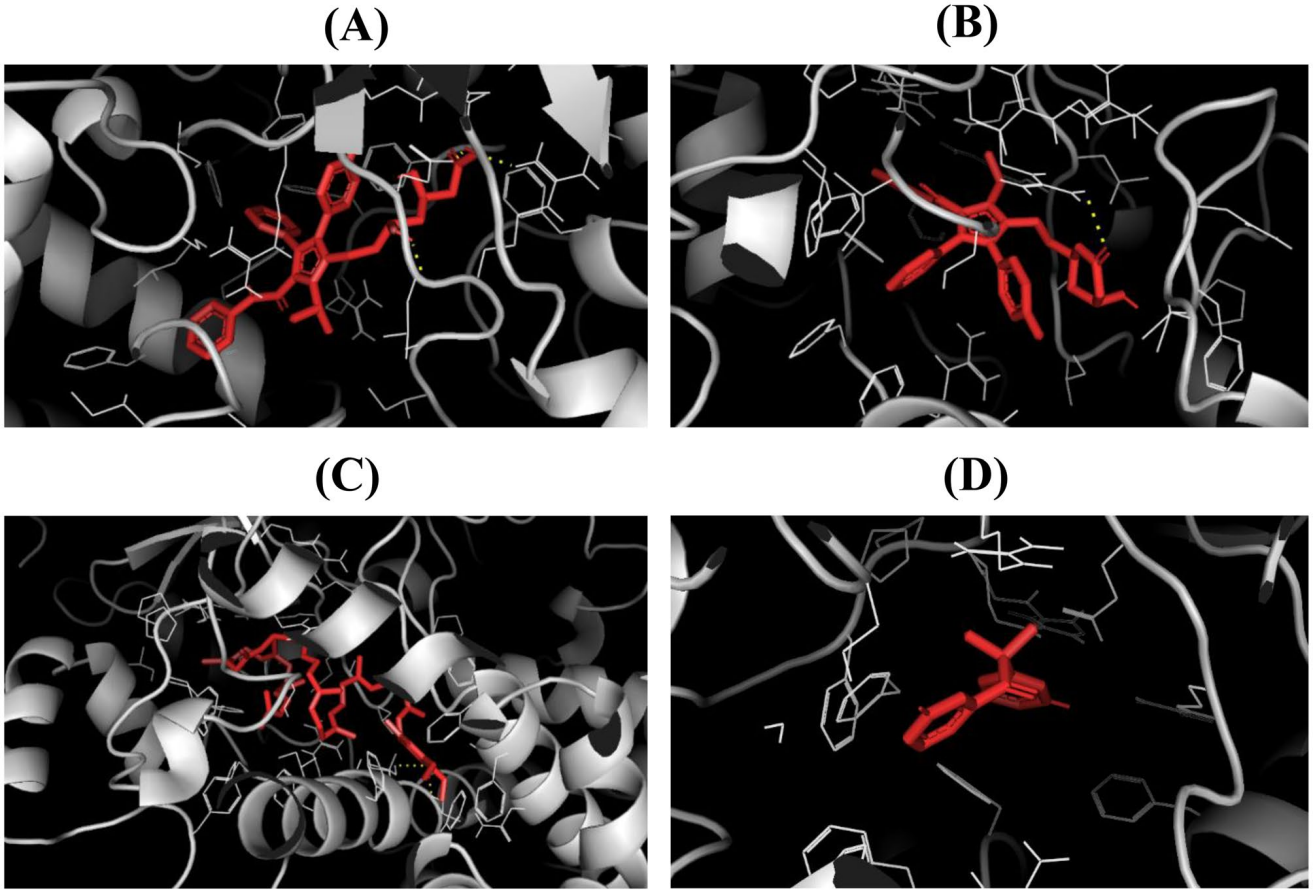
Docked pose of (**A**) ATO, (**B**) ATO lactone, (**C**) CoQ10, and (**D**) metyrapone within the cavity of CYP3A4. Abbreviations: ATO, atorvastatin; CoQ10, coenzyme Q10

Docking studies provided mechanistic insights into these experimental observations. ATO lactone showed the strongest affinity for CYP3A4 (–11.85 kcal/mol), followed by ATO (–10.26 kcal/mol) and CoQ10 (–10.05 kcal/mol), while metyrapone bound more weakly (–7.76 kcal/mol) (Fig. 7). ATO lactone and ATO formed extensive interactions with residues critical for CYP3A4 activity, while CoQ10 displayed comparable affinity, engaging hydrophobic and hydrogen-bonding residues such as Thr310, Leu373, and Phe304 (Fig. 7A–C).

**Fig. 8:**
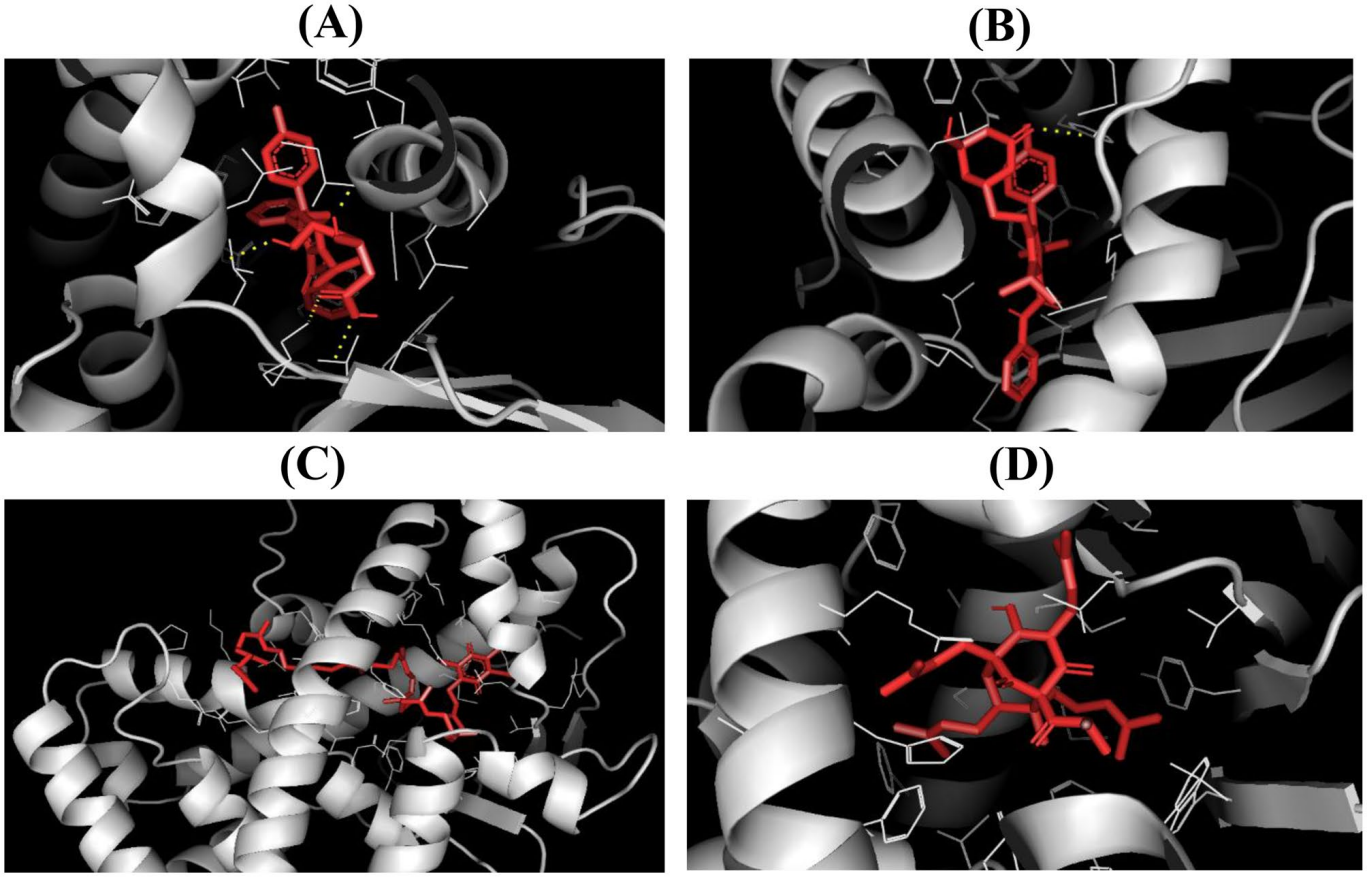
Docked pose of (**A**) ATO, (**B**) ATO lactone, (**C**) CoQ10, and (**D**) hyperforin within the cavity of PXR. Abbreviations: ATO, atorvastatin; CoQ10, coenzyme Q10; PXR, pregnane X receptor

For PXR, binding affinities were also strong, with ATO (–9.798 kcal/mol) and ATO lactone (–9.71 kcal/mol) exceeding hyperforin (–9.651 kcal/mol). CoQ10 showed a slightly weaker affinity (–7.748 kcal/mol) but still engaged hydrophobic residues within the ligand-binding pocket (Fig. 8A-D).

Importantly, these in silico findings align with experimental outcomes. CoQ10’s ability to engage CYP3A4 and PXR may help explain its capacity to mitigate ATO-induced oxidative stress, restore liver and kidney function, and preserve histological integrity. Thus, the biochemical, histological, immunohistochemical, and docking results converge on a common theme: CoQ10 offers significant hepatoprotection, with co-treatment consistently outperforming post-treatment.

## Discussion

Atorvastatin (ATO) disrupted hepatic redox balance and induced oxidative stress, as reflected by elevated lipid peroxidation and nitric oxide (NO) levels together with depletion of endogenous antioxidants. These findings are consistent with mitochondrial dysfunction and impaired electron transport, which promote excess reactive oxygen and nitrogen species [15, 26]. The marked reduction in GSH, CAT, and SOD confirmed that hepatocytes under statin pressure suffered weakened antioxidant defenses [27, 28]. Such redox collapse not only destabilizes mitochondrial membranes but also initiates downstream structural injury, helping to explain the vacuolation and disruption of hepatic cords observed histologically.

Coenzyme Q10 (CoQ10) supplementation effectively counteracted these disturbances by restoring electron flow and limiting radical leakage. As both a lipid-soluble antioxidant and respiratory chain cofactor, CoQ10 lowered MDA and NO while enhancing GSH, CAT, and SOD, thereby stabilizing redox signaling and improving hepatocellular resilience [29]. Protection was stronger with concurrent administration than with delayed treatment, indicating that early preservation of mitochondrial function prevents cumulative oxidative injury and preserves enzymatic defenses more effectively [30]. Importantly, this antioxidant recovery paralleled improvements in functional biomarkers, where co-treatment consistently outperformed post-treatment.

Atorvastatin also significantly impaired hepatic microsomal CYP3A1 activity, reducing it to ~ 29% of vehicle control in accordance with previous findings [31]. CoQ10 co-administration largely preserved CYP3A1 activity, whereas post-treatment partially restored it, consistent with the comparatively weaker protective effect of delayed supplementation. These findings indicate that CoQ10 maintains enzymatic activity associated with ATO metabolism, without requiring direct PXR measurement [32, 33].

The biochemical alterations translated into impaired liver and kidney function. Elevated ALT, bilirubin, and creatinine reflected mitochondrial dysfunction, impaired bile acid metabolism, and oxidative renal injury [34, 35]. These outcomes are attributable to mevalonate pathway inhibition, reduced endogenous CoQ10 synthesis, and weakened ATP generation [36]. Oxidative stress further aggravated hepatocellular leakage and bilirubin clearance defects, while renal oxidative damage impaired filtration [37, 38]. By restoring mitochondrial efficiency and stabilizing membranes, CoQ10 reduced hepatocyte leakage, improved bile handling, and lowered creatinine [39]. The stronger benefit of co-administration underscores the importance of sustaining mitochondrial integrity during statin exposure to prevent progressive organ injury.

Alterations in lipid metabolism further contextualize these findings. ATO decreased T. chol. and TAG through HMG-CoA reductase inhibition and enhanced LDL receptor activity, while raising HDL [40]. CoQ10 supplementation partially reversed these changes, restoring T. chol., TAG, and LDL-chol. compared with ATO alone [41, 42]. This rebound likely reflects recovery of mevalonate intermediates and mitochondrial acetyl-CoA flux, consistent with improved bioenergetics. The restoration of TAG levels observed in the combination treatment group can be attributed to the complementary effects of ATO and CoQ10 on lipid metabolism and mitochondrial function. While ATO exerts lipid-lowering effects, CoQ10 acts as a mitochondrial antioxidant, enhancing energy production and reducing oxidative stress [43]. Additionally, CoQ10 may influence the expression of key lipid-regulating genes, including peroxisome proliferator-activated receptor alpha (PPAR-α) and sterol regulatory element-binding protein 1c (SREBP-1c), which control lipid synthesis pathways [44].

Interestingly, the reversal of TAG levels was more pronounced with post-treatment, suggesting that discontinuation of statin-induced suppression, combined with mitochondrial support, allows for a more robust recovery of lipoprotein metabolism. By restoring mitochondrial function and limiting oxidative stress, CoQ10 likely contributes to rebalancing lipid homeostasis, leading to the observed normalization of TAG levels. Further studies are warranted to elucidate the precise molecular mechanisms underlying these effects.

Histopathology provided structural confirmation of these biochemical effects. ATO severely disrupted hepatic architecture, replacing normal lobular organization with central vein congestion, sinusoidal dilation, and hepatocellular vacuolation [45]. These lesions, graded on a semiquantitative 0–3 scale, reached severe (grade 3) under ATO and were consistent with mitochondrial dysfunction, lipid peroxidation, and apoptotic activation [46, 47]. CoQ10 co-treatment reduced vacuolation and preserved hepatic cords, lowering lesion scores to mild–moderate (grades 1–2). Kupffer cell infiltration suggested an adaptive protective response involving immune-mediated clearance of damaged cells [48]. Post-treatment showed only partial recovery: while sinusoidal dilation was reduced, central vein congestion and bile duct injury persisted, highlighting that once degenerative pathways are established, repair remains incomplete.

Bcl-2 expression served as a key indicator of the cellular defense against oxidative stress–mediated apoptosis. ATO treatment markedly downregulated Bcl-2, reflecting loss of mitochondrial integrity and activation of the Bax/Bak–cytochrome c–caspase pathway [49, 50]. In contrast, CoQ10 restored Bcl-2 expression, particularly under co-treatment conditions, suggesting stabilization of mitochondrial membranes and suppression of caspase-dependent apoptosis. The post-treatment regimen produced a weaker effect, consistent with limited reversal once apoptotic signaling is established. Overall, early CoQ10 administration effectively preserved mitochondrial function, mitigated oxidative stress, and reinforced anti-apoptotic defenses, and maintained CYP3A1 activity, thereby supporting hepatocyte survival and preventing irreversible cellular injury.

Molecular docking results offered mechanistic context. Both ATO and ATO lactone strongly bound to CYP3A4, with the lactone showing the highest affinity, which may delay metabolism and promote hepatic accumulation, aggravating oxidative stress and hepatotoxicity [51, 52]. Although PXR was not measured experimentally, docking indicated ATO binding to PXR, suggesting potential transcriptional activation of CYP3A4 that could enhance formation of reactive metabolites [53, 54]. By contrast, CoQ10 bound weakly to both CYP3A4 and PXR, implying that its protective effects stem not from direct enzyme modulation but from antioxidant and bioenergetic support [55]. This aligns with in vivo findings, where CoQ10 reduced oxidative damage while preserving CYP3A1 activity, despite lacking strong competition at enzyme-binding sites.

### Study limitations

Several limitations should be considered when interpreting these findings. We have now measured hepatic microsomal CYP3A1 activity, which provides direct experimental evidence of enzyme modulation by atorvastatin and the protective effects of CoQ10. However, PXR activity was not measured, and the link between CoQ10 and PXR-mediated CYP3A4 regulation remains inferred from docking analyses.

Molecular docking analyses offered mechanistic insight into interactions between ATO, its lactone metabolite, and the PXR–CYP3A4 system, but these predictions were not validated by pharmacokinetic or metabolism studies. Additionally, while the study assessed redox balance and Bcl-2 expression as indicators of mitochondrial function, direct experimental measurements of mitochondrial dysfunction (e.g., ATP content, mitochondrial ROS, membrane potential, or respiration assays) were not included. Such experiments would provide a more direct assessment of mitochondrial impairment.

The study also focused on CoQ10 supplementation at a single dose and did not explore dose-dependent effects, which would be important for optimizing therapeutic efficacy. Further investigations are needed to examine additional mechanistic pathways, including potential effects of CoQ10 on lipid metabolism and drug–nutrient interactions.

Finally, translation to humans should be approached with caution due to interspecies differences in drug metabolism and CoQ10 bioavailability. Despite these limitations, the study provides preclinical evidence demonstrating that CoQ10 preserves CYP3A1 activity and mitigates ATO-induced hepatotoxicity, offering a foundation for future mechanistic, pharmacokinetic, and translational studies to improve statin safety.

## Conclusion

This study demonstrates that atorvastatin (ATO) disrupts hepatic redox homeostasis and antioxidant defenses, leading to mitochondrial dysfunction, activation of apoptotic pathways, and functional impairment of hepatic CYP3A1 activity, with associated hepatic alterations. Coenzyme Q10 (CoQ10) attenuated these effects by supporting mitochondrial function, restoring redox balance, preserving anti-apoptotic signaling, and partially maintaining CYP3A1 activity. Notably, protective effects were more pronounced when CoQ10 was administered concurrently with ATO than when given after exposure, underscoring the importance of intervention timing.

Collectively, these findings suggest that CoQ10 can modulate metabolism- and mitochondria-linked adverse effects of ATO in a preclinical setting. However, further studies addressing PXR-related pathways, pharmacokinetics, dose optimization, and translational relevance are required before these observations can be extrapolated to clinical practice.

## Data Availability

Data will be made available upon request.

## Abbreviations

ATO: Atorvastatin
Bcl-2: B-Cell Lymphoma 2
CAT: Catalase
CoQ10: Coenzyme Q10
Co-tx: Co-Treatment
CYP3A4: Cytochrome P450 3A4
D bil: Direct Bilirubin
DILI: Drug-Induced Liver Injury
MDA: Malondialdehyde
Post-tx: Post-Treatment
PXR: Pregnane X Receptor
RNS: Reactive Nitrogen Species
ROS: Reactive Oxygen Species
SOD: Superoxide Dismutase
T bil: Total Bilirubin
mPTP: Mitochondrial Permeability Transition Pore
